# Development of a Method for Producing oxLDL: Characterization of Their Effects on HPV-Positive Head and Neck Cancer Cells

**DOI:** 10.3390/ijms232012552

**Published:** 2022-10-19

**Authors:** Alessandro Scalia, Nadège Kindt, Anne Trelcat, Amandine Nachtergael, Pierre Duez, Fabrice Journé, Stéphane Carlier

**Affiliations:** 1 Department of Cardiology, UMONS Research Institute for Health Sciences and Technology, University of Mons (UMONS), 7000 Mons, Belgium; 2 Department of Cardiology, Centre Hospitalier Universitaire Ambroise Paré, 7000 Mons, Belgium; 3 Department of Clinical and Experimental Oncology, Institut Jules Bordet, Université Libre de Bruxelles, 1000 Brussels, Belgium; 4 Department of Human Anatomy and Experimental Oncology, UMONS Research Institute for Health Sciences and Technology, University of Mons (UMONS), 7000 Mons, Belgium; 5 Department of Therapeutic Chemistry and Pharmacognosy, University of Mons (UMONS), 7000 Mons, Belgium

**Keywords:** oxLDL, HNCC, HPV, LOX-1, CD36, cell migration

## Abstract

Cardiovascular diseases (CVD) and cancers are the two main causes of death worldwide. The initiation and progression of atherosclerosis is, in large part, caused by oxidized low-density lipoproteins (oxLDL); interestingly, oxLDL may also play a role in cancer cell metabolism and migration. As oxLDL are generally obtained by tedious ultracentrifugation procedures, “*home-made*” oxLDL were obtained by *(i)* applying a purification kit to isolate LDL and VLDL from human plasma; *(ii)* isolating LDL from VLDL by gel permeation chromatography (GPC); and *(iii)* oxidating LDL through CuSO_4_ incubation. On three HPV-positive head and neck cancer cells (HNCC) (93VU-147T, UM-SCC47, and UPCI-SCC154), cell migration was assessed using Boyden chambers, the Wnt/ß-catenin pathway was analyzed by Western Blotting, and the expression of two oxLDL receptors, LOX-1 and CD36, in response to oxLDL exposure, was analysed by immunofluorescence. Our data indicate: (a) a non-significant difference between reference and “*home-made*” oxLDL; (b) a decreased migration, parallel to an inhibition of the ß-catenin pathway; and (c) an increase of CD36 and LOX-1 expression in all HNCC. In conclusion, we successfully produced oxLDL. Our results demonstrate a decrease in HNCC migration after oxLDL exposure, and an increased expression of LOX-1 and CD36 associated with lipid uptake.

## 1. Introduction

Cardiovascular diseases (CVD) and cancers are the two main causes of death worldwide, representing around 17.9 million and 10 million deaths per year, respectively, according to the World Health Organization (WHO) [1,2]. Head and neck cancers (HNC) represent approximatively 900,000 new diagnoses and 400,000 deaths worldwide [3]. It is well known that CVD and cancers share many comorbidities and risk factors, notably tobacco use, alcohol consumption, unhealthy diets, or physical inactivity.

A pro-inflammatory state with neoangiogenesis and oxidative stress seems to be the key link between them. Atherosclerosis, the major actor in CVD, damages both small and large vessels and involves several interrelated processes, including lipid disturbances, platelet activation, thrombosis, endothelial dysfunction, inflammation, oxidative stress, vascular smooth cell activation, altered matrix metabolism, and extracellular matrix remodeling.

During the first steps of atherosclerosis, low density lipoproteins (LDL) move into the altered subendothelium where they are oxidized by macrophages and smooth muscle cells (SMC). OxLDL transform macrophages into foam cells that release growth factors and cytokines [4]. In addition, endothelial cells (EC), activated by oxLDL, overexpress some cytokines, such as the Macrophage Colony-Stimulating Factor (M-CSF), to attract additional monocytes and differentiate them into macrophages [5].

Scavenger receptors, including the cluster of differentiation 36 (CD36), the lectin-like oxidized low-density lipoprotein receptor-1 (LOX-1), the scavenger receptor type A (SR-A) and the scavenger receptor class B type 1 (SRB-1), which are strongly expressed on endothelial cells (EC), smooth muscle cells (SMC), and fibroblasts, promote the internalization of oxLDL in macrophages, switching them to foam cells [6]; macrophages expressing such scavenger receptors on their surface present a decreased migration potential and are then retained within the plaque [7]. Furthermore, oxLDL fixation on fibroblasts scavenger receptors leads to an increased production of collagen in the plaque, allowing them to catch even more LDL [8]. Lastly, scavenger receptors induce the formation of reactive oxygen species (ROS) and SMC proliferation that promote some of the previously described alterations [9].

In cancer, inflammation occurs by the infiltration of immune cells, including macrophages, T cells, or natural killer cells, that secrete large amounts of inflammatory cytokines, pro-angiogenic factors, and ROS, in the tumor microenvironment [9]. Some studies have already implied oxLDL in cancer; indeed, an increased level of serum oxLDL is observed in patients with colorectal, breast, or ovarian cancer [10,11] and animal experiments indicate an increased incidence of prostate, colon, and liver cancers upon fat-enriched diets [12]. Additionally, a depletion in LOX-1 receptors protects against the apparition of new cancers among several lineages, e.g., breast and cervical cancers and hepatocellular carcinoma [13], whereas an increased LOX-1 expression has been reported in gastric cancer tissues [14].

Our team recently studied the migration profile of head and neck cancer cells (HNCC) with Human Papilloma Virus (HPV) negative status, after exposure to commercial oxLDL. We demonstrated an increased expression of LOX-1 and CD36 receptors in HNCC after oxLDL exposure, and a decrease in cell migration via changes in the CD36/b-catenin pathway [15].

In the present study, we propose to produce well-characterized oxLDL in order to control and standardize their production. These “*home-made*” oxLDL will be characterized by gel electrophoresis and compared with a commercially available oxLDL batch on one HNCC (93VU-147T) cell line; they will be applied in two additional HPV-positive HNCC to study whether their effects on cell migration involve the CD36 scavenger receptor.

## 2. Results

### 2.1. Control of the Quality of LDL Oxidation by Agarose Gel Electrophoresis

**Figure 1:**
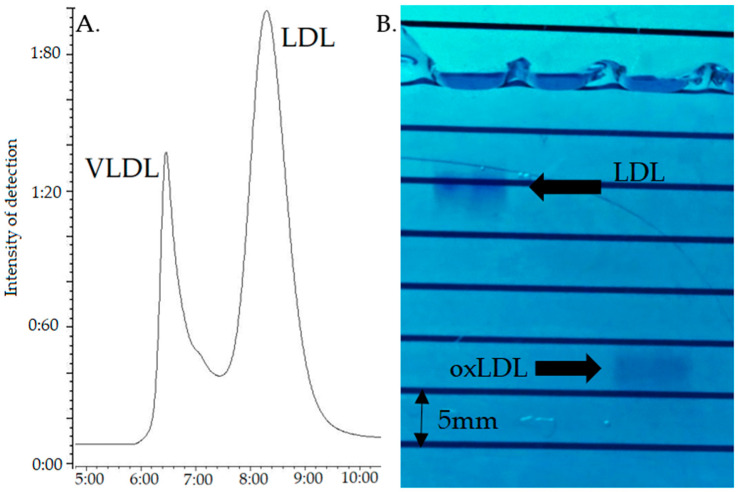
(**A**) Typical GPC chromatogram of the LDL/VLDL extract obtained by selective precipitation from human plasma. (**B**) Migration pattern of LDL (left) and Cu^++^-treated LDL (oxLDL, right) migration on a 0.5% agarose gel electrophoresis, after 60 min running at 70 V.

In the applied electrophoresis conditions (Section 2.1), native LDL migrated up to 10.6 mm, while Cu^++^-freshly oxidized LDL migrated up to 26.6 mm (Figure 1B).

### 2.2. OxLDL Internalization in HNCC

**Figure 2:**
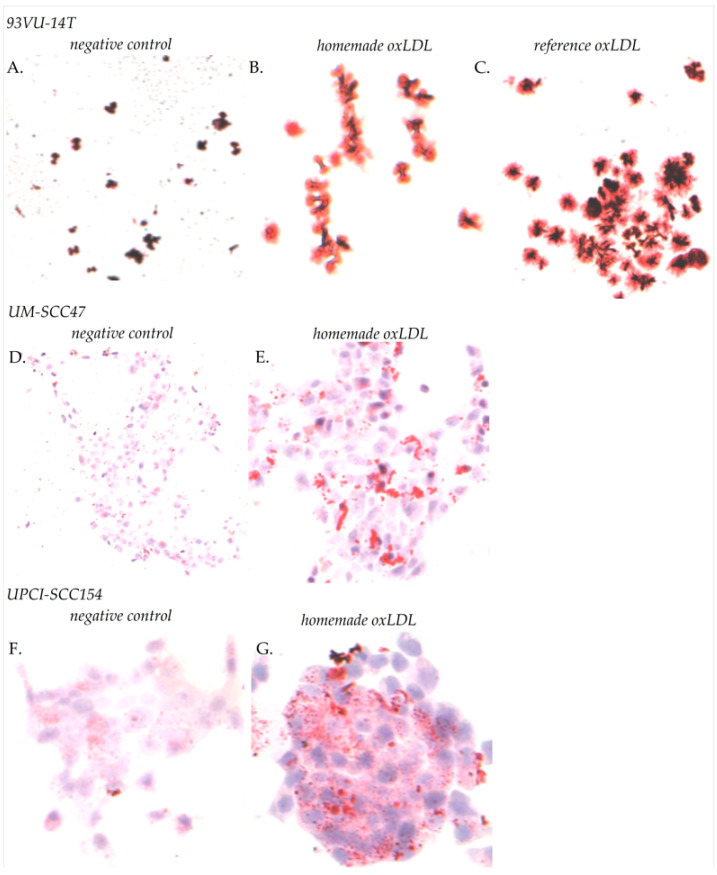
(**A**) Oil red O staining of HNCC cells, after 48 h of treatment (×40 magnification). 93VU-147T cells without exposure to oxLDL (negative control); (**B**) with exposure to 30 µg/mL home-made oxLDL; or (**C**) to 30 µg/mL reference oxLDL. (**D**) UM-SCC47 cells without exposure to oxLDL; and (**E**) with exposure to 30 µg/mL home-made oxLDL. (**F**) UPCI-SCC154 cells without exposure to oxLDL; and (**G**) with exposure to 30 µg/mL home-made oxLDL.

OxLDL uptake in cancer cells was detected after Red Oil staining. On 93VU-147T cells, we compared our “*home-made*” oxLDL with reference oxLDL: 47.7% of cells showed an uptake with our oxidized LDL against 42.4% with commercially available oxLDL (48 h exposure; Figure 2B,C). Negative control shows 12.2% uptake (Figure 2A) (*home-made* oxLDL vs. negative control, *t*-test, *p* = 0.007; reference oxLDL vs. negative control, *t*-test, *p* = 0.002). No significant difference was highlighted between our home-made and reference oxLDL (*t*-test, *p* = 0.483).

On UM-SCC47 and UPCI-SCC154 cells, home-made oxLDL induced an uptake of 46.3% (negative control, 12.3%) (48 h exposure; Figure 2D,E) and 64.8% (negative control, 10.3%), respectively (48 h exposure; Figure 2F,G) (UM-SCC47 *home-made* oxLDL vs. negative control, *t*-test, *p* = 0.006; UPCI-SCC154 *home-made* oxLDL vs. negative control, *t*-test, *p* = 0.002).

### 2.3. Scavenger Receptors Expression on HNCC upon Exposure to oxLDL

**Figure 3:**
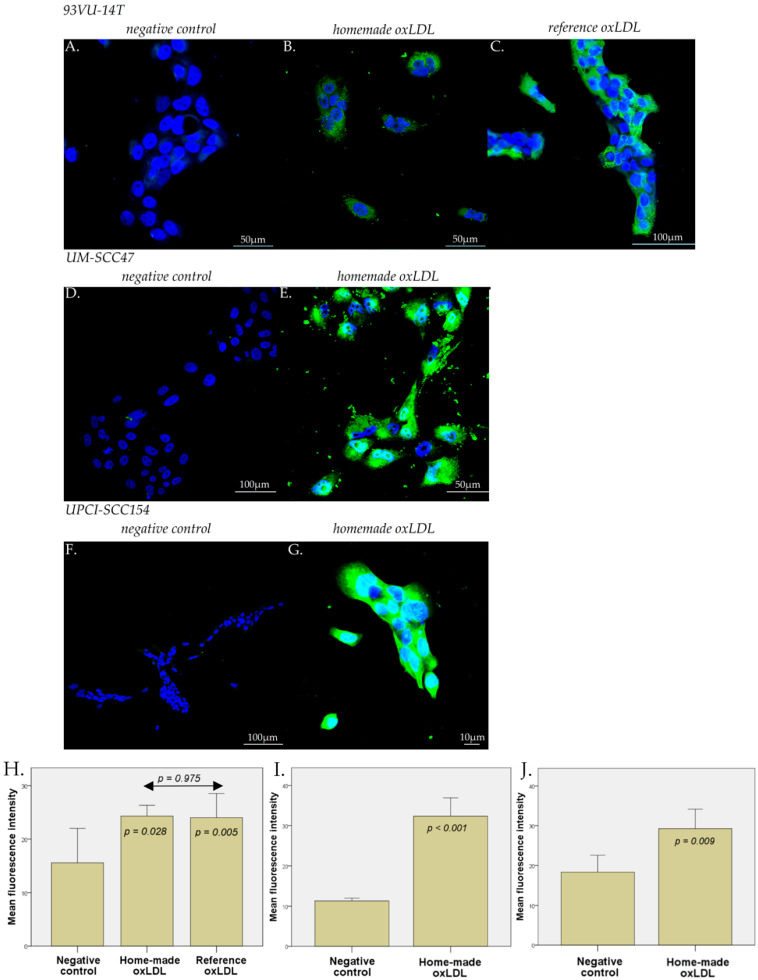
CD36 expression in 93VU-147T, UM-SCC47, and UPCI-SCC154 cell lines with or without oxLDL treatment (30 µg/mL, 48 h). Representative images and computed mean fluorescence intensities (MFI; 2 biological replicates in triplicate). (**A**,**H**) CD36 expression in 93VU-147T not exposed to oxLDL (*n* = 10); (**B**,**H**) or exposed to home-made oxLDL (*n* = 10); or (**C**,**H**) reference oxLDL (*n* = 10). (**D**,**I**) CD36 expression in UM-SCC47 cells without oxLDL (*n* = 10); or (**E**,**I**) exposed to home-made oxLDL (*n* = 10). (**F**,**J**) CD36 expression on UPCI-SCC154 cells without oxLDL (*n* = 10); or (**G**,**J**) with home-made oxLDL (*n* = 10).

**Figure 4:**
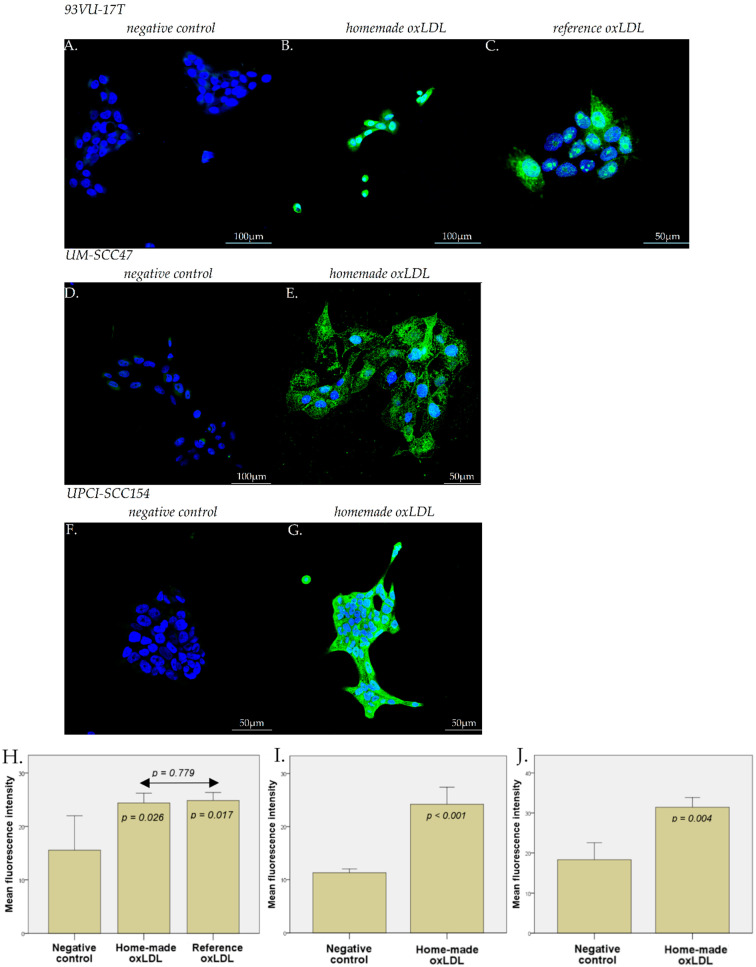
LOX-1 expression on 93VU-147T cells with (**A**) no treatment, with (**B**) home-made oxLDL, or (**C**) reference oxLDL. (**H**) Chart representing LOX-1 expression on 93VU-147T cells with a significant expression both with our home-made oxLDL (*p* = 0.026) and reference oxLDL (*p* = 0.017) compared to control, but no difference between home-made and reference oxLDL (*p* = 0.779) (*n* = 10). LOX-1 expression on UM-SCC47 cells with (**D**) no treatment or with (**E**) home-made oxLDL, and (**I**) the chart representing the significantly higher expression of LOX-1 (*n* = 10). (**F**) LOX-1 expression on UPCI-SCC154 cells with no treatment or with (**G**) home-made oxLDL, and (**J**) the chart representing the significantly higher expression of LOX-1 (*n* = 10).

Exposure of HNCC to oxLDL for 48 h results in a significant increase in both CD36 and LOX-1 expression, equally in the three tested cell lines (Figure 3 and Figure 4). The mean fluorescence intensity (MFI), corresponding to the CD36 expression in 93VU-147T cells in negative control conditions (no treatment), was 15.54, compared with 24.29 for home-made oxLDL and 24.00 for reference oxLDL (Figure 3A–C,H; *t*-test, *p* = 0.028 and *p* = 0.005, respectively against negative control). The LOX-1 MFI in the 93VU-147T cells negative control (no treatment) was 14.97 compared with 24.39 for home-made oxLDL and 24.85 for reference oxLDL (Figure 4A–C,H; *t*-test, *p* = 0.026 and *p* = 0.017, respectively). No significant difference was highlighted between home-made oxLDL and reference oxLDL (*p* = 0.975 for CD36 expression and 0.779 for LOX-1 expression).

On UM-SCC47 cells and UPCI-SCC154 cells, the CD36 MFI were 32.37 (negative control, 11.70) and 29.26 (negative control, 18.31), respectively, and the LOX-1 MFI were 24.19 (negative control, 12.15) and 31.43 (negative control, 16.40), respectively (Figure 3D–J and Figure 4D–J) (*t*-tests, *p* < 0.01).

### 2.4. Effect of oxLDL on HNCC Migration and Involvement of CD36

**Figure 5:**
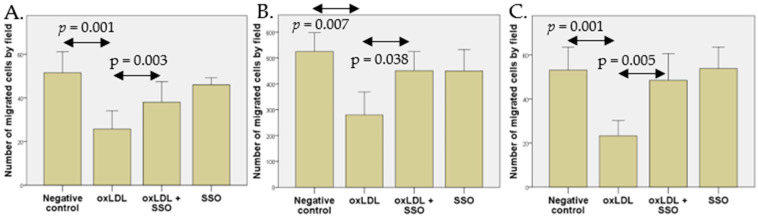
Migration of 93VU-147T, UM-SCC47, and UPCI-SCC154 cell lines exposed to oxLDL (30 µg/mL, 48 h). (**A**) Number of migrated 93VU-147T (*t*-test, *p* = 0.001); (**B**) UM-SCC47 (*t*-test, *p* = 0.007); and (**C**) UPCI-SCC154 (*t*-test, *p* = 0.001) cells by x10 magnification field under oxLDL and SSO conditions.

Migration assays indicate a decreased migration of cells after 48 h oxLDL exposure (Figure 5). These decreased migrations were overcome by 4 h pre-incubation with SSO, a CD36 inhibitor, demonstrating the involvement of this receptor in the migration ability of HPV-positive HNCC. No significant difference was highlighted between the treatments oxLDL + SSO and SSO alone (Figure 5). Similar results were obtained in the three HNCC, suggesting that CD36 inhibition by SSO does restore the cell migration inhibited by oxLDL.

For the 93VU-147T (Figure 5A), UM-SCC47 (Figure 5B), and UPCI-SCC154 (Figure 5C) cell lines, an average of 25.7 (vs. 51.5 in the non-treated condition), 279.7 (vs. 524.9 in control), and 23.3 (vs. 53.1 in control) cells by field had migrated upon oxLDL exposure, respectively. The SSO pre-incubation resulted in a significantly lower effect of oxLDL exposure, the cell migration increasing to 38.0, 450.7, and 48.5 cells by field, respectively.

### 2.5. Involvement of the Wnt/b-Catenin Pathway on the Decrease of HNCC Migration

**Figure 6:**
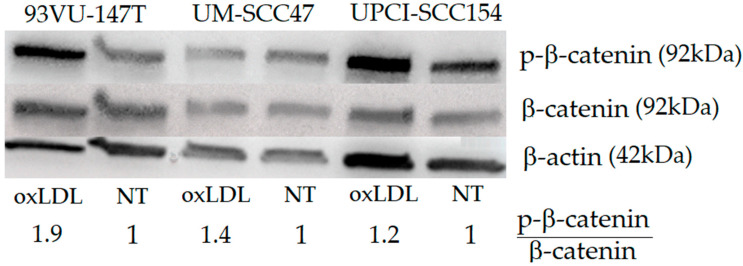
Western blot membrane showing the expression of P-b-catenin, b-catenin, and b-actin with and without oxLDL treatment (30 µg/mL, 48 h). NT = no treatment, cells not exposed to oxLDL.

Western blotting demonstrated an increased P-b-catenin/b-catenin ratio upon oxLDL exposure unequally on the three cell lines (Figure 6). Indeed, the 93VU-147T cells had a greater change in P-b-catenin/b-catenin ratio (1.9), compared to the UM-SCC47 and UPCI-SCC154 cells, for which the ratio was increased to 1.4 and 1.2, respectively.

## 3. Discussion

As oxLDL are among the main actors in the initiation and progression of atherosclerosis, it is necessary to produce oxLDL for studies involving their biological effects. The method most often applied to isolate lipoproteins producing oxLDL relies on ultracentrifugation, at around 100.000 g during 20–24 h with potassium bromide (KBr), in order to modify the density of the solution at 1.063 g/mL to isolate lipoproteins [16,17]. Some older studies even used a higher relative centrifugal force at 250.000–280.000 g during 24 to 48 h [18,19]. However, these methods are laborious in daily practice, requiring special and large equipment and long running times [20]. Therefore, we developed a method easier to implement, that only requires material usually available in most laboratories. All the steps can be performed within a week even for a large production, requiring an initial investment of some 2000€. After selective precipitation from human plasma of a mixture LDL/VLDL, size exclusion liquid chromatography was used to isolate each of these lipoprotein classes, but also to assess the purity of the isolated LDL fraction by verifying the absence of other lipoproteins.

CuSO_4_ is commonly used for LDL oxidation because it is easy to use, cheap, and steady. However, the resulting oxidation is “aggressive”, and may not be representative of in vivo oxidations. The intensity of oxidation depends on CuSO_4_ concentration, the duration and temperature of incubation (increasing the temperature from 21 °C to 37 °C severely increases oxidation), and LDL concentration [21]. Thus, it is extremely important that we standardized a process of mild LDL oxidation. It could be interesting to implement an even softer oxidation method to fit with real life situations. Indeed, it has been shown on M0 macrophages that the strength of oxidation influences the polarization to M1 or M2 macrophages [22]. Additionally, Vlaminck et al. compared two models of LDL oxidation, the classical CuSO_4_ oxidation (oxLDL) and a more physiological hydrogen peroxide-myeloperoxidase-based peroxidation that yields MoxLDL. They showed that copper oxidation induces biochemical alterations on both lipids and proteins, while myeloperoxidation induces mainly alterations on proteins; also, on THP-1-derived macrophages, oxLDL and MoxLDL induced different cell death pathways [23]. Therefore, a majoring effect on HNCC of our LDL Cu^++^ oxidation cannot be excluded, and our levels of protein oxidation should be quantitatively assessed, e.g., by an ELISA method. However, we obtained similar biological data by comparing reference and home-made oxLDL, which suggests an acceptable rate of oxidation in our production.

The results obtained in this work on HPV-positive HNCC are in line with our previous results, obtained with reference to oxLDL [15] on HPV-negative HNCC (Detroit 562, UPCI-SCC-131 and FaDu), that indicated similar scavenger receptors expression and migration profiles.

However, interestingly, recent papers suggest that high-risk HPV may increase the risk of atheromatous plaque. The mechanism remains unclear, but it was proposed that the systemic inflammation induced by HPV could promote atherosclerosis lesions. The pro-inflammatory effects of HPV and the release of extracellular vesicles by HPV-transformed cells are well documented in the scientific literature [24]. Data from the National Health and Nutrition Examination Survey on U.S. women indicate a strong association between the presence of HPV DNA on vaginal swab and a prior history of myocardial infarction or stroke [25]. On the other hand, Lawson et al. identified HPV in 55% of atheromatous coronary arteries from deceased donors [26], while Joo et al. described an elevated long-term cardiovascular risk among healthy young Korean women infected with high-risk [27]. Thus, it is highly probable that there is a relation between HPV status and atherosclerosis, but further studies need to be implemented to understand the mechanisms involved.

We recognize some limitations of our work, e.g., that different intensities of copper oxidation or myeloperoxidation could be tested on HNCC in order to observe potential variability in scavenger receptors expression or cell migration

Moreover, in vitro results on 2-dimensional (2D) models are not able to mimic the microenvironment of cancers. Some authors have chosen to study HNCC effects on a novel 3-dimensional (3D) model, which comes closest to the in vivo situation. Miserocchi et al. showed that culturing oropharyngeal squamous cell carcinoma (OSCC) on a 3D biomimetic collagen-based scaffold induced different collagen fiber organization, and induced an increased expression of markers related to epithelial–mesenchymal transition (EMT) associated to a different cell migration behavior independently of HPV status. They demonstrated that LOX-1 expression was the most upregulated marker in 3D cultures, approximately ten-fold higher than in monolayer cultures [28]. In parallel, Engelmann et al. recently worked on primary HNCC from patient’s tumorectomy cultured in a 3D organotypic co-culture model (3D-OTC) that maintain the architecture and cell composition to mimic individual tumors in their entirety, and to test individual long-term tumor responses to standard treatment or targeted therapies [29].

Future experiments on the involvement of LOX-1 and CD36 receptors in migration might be interesting in determining an eventual synergistic effect. It would also be interesting to observe a possible effect of the LDL oxidation level on cancer cell migration and scavenger receptor expression, e.g., by changing the CuSO_4_ incubation conditions, or by applying myeloperoxidation or a superoxide anion generator. Finally, it would be interesting to reproduce these experiments on a 3D-model in order to mimic the in vivo tumor environment.

In conclusion, we developed an original method to produce oxLDL and demonstrated that oxLDL exposure of HPV-positive HNCC enhanced their lipid uptake and increased the expression of CD36 and LOX-1 scavenger receptors, while decreasing their migration capacity.

## 4. Material and Methods

### 4.1. oxLDL Production

Commercial oxLDL (Invitrogen, ThermoFisher Scientific, Waltham, MA, USA) were used as reference comparators for the “*home-made*” oxLDL. On 10 mL human plasma (Croix-Rouge, Bruxelles, Belgium), we applied a purification kit (Cell Biolabs Inc., San Diego, CA, USA) based on a precipitation and centrifugation step to isolate a 1 mL fraction containing LDL and Very Low Density Lipoproteins (VLDL). This kit is based on dextran sulfate to selectively precipitate LDL and VLDL without the need for ultracentrifugation. LDL were isolated from VLDL by high performance liquid chromatography (system HPLC 626-DAD 2996, Waters™, Framingham, MA, USA), by injecting 150 µL of the purified LDL-VLDL fraction in an aqueous size exclusion chromatography column (Shodex PROTEIN KW-804, 8.0 mm i.d. × 300 mm, couple to a KW-G 6B column, 6.0 mm i.d. × 50 mm). The chromatographic separation was achieved at 30 °C at a constant flow rate of 1 mL/min using Phosphate-Buffered Saline 1X (PBS, Gibco Life Technologies, Paisley, UK) as the mobile phase. The DAD detection wavelength was set at 280 nm.

Under these conditions, VLDL and LDL eluted at about 6.6 and 8.6 min (Tmax), respectively (Figure 1A); given the non-baseline resolution, the LDL peak was recovered after complete elution of the VLDL, i.e., starting at about 8.0 min. This LDL fraction (3 mL) was oxidized by incubation with 5 µM CuSO_4_ (VWR international, Avantor, Radnor, PA, USA) for 20 h at 37 °C, the reaction being stopped with 0.2 mM EDTA. The oxLDL was then dialyzed against PBS containing 1 mM EDTA during 24 h at 4 °C, as described by Vrieling et al., using the Slide-A-Lyzer™ MINI dialysis 20K MWCO device (ThermoFischer Scientific, Waltham, MA, USA) [30], with 2 buffer replacement steps.

The quality of oxidation was assessed by 0.5% agarose gel electrophoresis running for 60 min at 70 Volt [31], the bands being visualized with Coomassie Blue staining (SimplyBlue™ SafeStain, Invitrogen™, Carlsbad, CA, USA) during 24 h followed by 48 h deionized water washing. As electronegativity increases with oxidation, native LDL migrate under these conditions at approximatively 11 mm against 12 to 30 mm for the oxLDL, depending on their oxidation status [21].

### 4.2. Cell Culture

The 93VU-147T cell line was grown in Dulbecco’s Modified Eagle Medium (DMEM, Lonza, Verviers, Belgium) supplemented with 10% fetal bovine serum (FBS), 2% L-Glutamine, and 1% Penicillin/Streptomycin (Gibco Life Technologies, Paisley, UK).

The UM-SCC47 was cultured in DMEM supplemented with 10% FBS, 2% L-Glutamine, 1% Penicillin/Streptomycin, and 1% non-essential amino acids (Gibco Life Technologies, Paisley, UK).

The UPCI-SCC154 cell line was grown in Minimum Essential Medium (MEM, Gibco Life Technologies, Paisley, UK) supplemented with 10% FBS, 2% L-Glutamine, 1% Penicillin/Streptomycin, and 1% non-essential amino acids (Gibco Life Technologies, Paisley, UK).

Routine cell culture was carried out at 37 °C in a humidified cell incubator under 5% CO_2_.

### 4.3. Oil Red O Staining

The amount of oxLDL internalization in cancer cells was evaluated by Oil Red staining. HNCC were plated on sterile round glass coverslips in a 12-wells dish at 60,000 cells/well during 24 h. For 93VU-147T cells, the medium was replaced by fresh medium with 30 µg/mL reference oxLDL, or 30 µg/mL home-made oxLDL, or fresh medium alone, for 48 h. For UM-SCC47 and UPCI-SCC154 cells, the medium was replaced by fresh medium with 30 µg/mL home-made oxLDL or fresh medium alone. The medium used was prepared with 10% Charcoal Stripped FBS (Labconsult SA-NV, Brussels, Belgium), which is delipidated with active carbon, instead of standard, decomplemented FBS. Cells were fixed with 4% paraformaldehyde in Dulbecco’s Phosphate-Buffer (DPBS, Lonza, Verviers, Belgium), then rinsed with DPBS and stained with Oil Red O (Merk Sigma, Darmstadt, Germany) for 15 min at room temperature (RT). After three rinsing steps with distilled water, cells were mounted with Aquatex^®^ (Merk Sigma, Darmstadt, Germany) and observed by phase-contrast microscopy using a Zeiss axioplan microscope equipped with a color charge-coupled device (CCD) camera (ProgRes C10plus, Jenoptik, Jena, Germany). Five fields were captured at ×10 magnification. Total cells and cells having accumulated lipids were counted using ImageJ (Research Services Branch of the National Institute of Health, NIH, Bethesda, MD, USA) [32].

### 4.4. Immunofluorescence Microscopy

HNCC were plated on sterile round glass coverslips in a 12-well dish at 60,000 cells/well during 24 h. For 93VU-147T cells, the medium was replaced by fresh stripped medium with 30 µg/mL reference oxLDL, or 30 µg/mL home-made oxLDL, or fresh stripped medium alone, for 48 h. For UM-SCC47 and UPCI-SCC154 cells, the medium was replaced by fresh stripped medium with 30 µg/mL home-made oxLDL or fresh stripped medium alone. After 48 h, cells were fixed with 4% paraformaldehyde in PBS for 10 min at 4 °C and 5 min at RT.

For the anti-CD36 primary antibody (Merk Sigma, Darmstadt, Germany), the fixed cells were preincubated for 20 min at RT in a solution containing 2% Bovine Serum Albumin (BSA) in PBS to prevent non-specific adsorption of immunoglobulins. Then, anti-CD36 was diluted at 1/100 in the same solution and applied on cells overnight at 4 °C.

For the anti-LOX-1 primary antibody (ThermoFisher Scientific, Waltham, MA, USA), the fixed cells were pre-incubated for 1 h at RT in a solution containing 5% Normal Goat Serum (NGS)/Triton-X100 0.3% in PBS. Then, anti-LOX-1 was diluted at 1/1000 in the same solution and applied on cells overnight at 4 °C.

After several rinses with PBS, cells were treated with an anti-rabbit IgG antibody coupled with Alexa 488 for 30 min (ThermoFisher Scientific, Waltham, MA, USA), diluted in their respective solution.

After the final rinses with PBS and deionized water, the coverslips were mounted onto glass slides using a commercial anti-fading medium (ProLong™ Gold antifade reagent with DAPI, Invitrogen, ThermoFisher Scientific, Waltham, MA, USA). Confocal microscopy observations were carried out using an Olympus FV1000D laser scanning inverted microscope (LD559) (Olympus, Tokyo, Japan).

### 4.5. Cell Migration Assay

A Boyden Chamber assay was used to determine cell migration. A 24-wells plate containing fresh complete stripped medium (as described in Section 2.2) was used as lower chamber and cell culture inserts (Falcon™ Cell Culture Inserts, ThermoFisher Scientific, Waltham, MA, USA) containing fresh serum-free medium were used as upper chambers. Lower and upper chamber were separated by a polycarbonate membrane (8 µm pore size). After 1 h of membrane rehydration with serum-free medium, 93VU-147T and UPCI-SCC154 cells were plated with 300,000 cells/inserts, and UM-SCC47 cells were plated at 120,000 cells/insert. The following day, the lower chamber was replaced with a complete stripped medium with or without 30 µg/mL home-made oxLDL. On 93VU-147T cells, an additional condition with 30 µg/mL of reference oxLDL was tested.

After 48 h, cells were wiped from the upper surface of the cell culture inserts with a cotton-tipped swab and the migrating cells were stained with crystal violet, after fixation with 4% paraformaldehyde. Migration was assessed by counting migrated cells over five microscopic fields (×10 magnification) using a Panthera C2 Classic Motic microscope (Motic^®^Europe, Barcelona, Spain).

An inhibition of CD36 was realized with sulfosuccinimidyl oleate (SSO), an irreversible inhibitor of CD36 that leads to a reduction in oxLDL uptake [33]. Cells were seeded in cell culture inserts as previously described. The following day, cells were treated with 1 µM of SSO (Cayman chemical, Ann Arbor, MI, USA) for 4 h in a serum-free medium. Then, the medium containing SSO was replaced by fresh serum-free medium. The medium in the lower chambers was replaced with completed stripped medium with or without 30 µg/mL oxLDL. After 48 h, the cells were fixed with 4% paraformaldehyde and stained with crystal violet. The numbers of migrated cells were evaluated over five microscopic fields.

### 4.6. Western Blotting

Given its importance in cell motility and migration [34], the Wnt/b-catenin pathway was further evaluated. Once cells reached 70–80% confluence on T75 cell culture flask, (ThermoFisher Scientific, Waltham, MA, USA), 93VU-147T, UM-SCC47, and UPCI-SCC154 cells were treated, or not, with 30 µg/mL home-made oxLDL for 48 h. Afterward, a lysis buffer (M-PER Mammalian Protein Extraction Reagent, ThermoFisher Scientific, Waltham, MA, USA) supplemented with protease and phosphatase inhibitors (Halt Protease and Phosphatase inhibitor cocktail, ThermoFisher Scientific, Waltham, MA, USA) were applied to lyse cells that were then scraped and centrifuged at 13,000 rpm for 10 min to collect protein lysates. Protein concentrations were assessed by a BCA protein assay (Pierce, ThermoFisher Scientific, Waltham, MA, USA) using bovine serum albumin as the standard. An amount of 25 µg of extracted proteins were loaded on 4–20% Mini-PROTEAN TGX gels (PAGE-SDS) (Bio-Rad Laboratories, München, Germany) and separated using electrophoresis at 120 V during 1 h. Then, proteins in the gel were electrotransferred onto nitrocellulose membranes (iBlot^®^ Dry Blotting System, Life Technologies-Invitrogen, Ghent, Belgium).

After a preincubation with PBS/Tween 0.1%/Milk powder 5% to prevent non-specific adsorption of immunoglobulins, the immunodetection was performed using an anti-β-catenin antibody (1/1000), an anti-P-β-catenin antibody (to detect the phosphorylation of β-catenin at the S675; 1/1000) (Cell Signaling, Danvers, MA, USA), and an anti-actin antibody (1/1000) (Pierce, ThermoFisher Scientific, Waltham, MA, USA).

A peroxidase-labeled anti-rabbit IgG antibody (1/5000) (Amersham Pharmacia Biotech, Roosendaal, the Netherlands) and a peroxidase-labeled anti-mouse IgG antibody (1/2000) were used as the secondary antibodies. The bound peroxidase activity was detected using the SuperSignal^®^ West Pico Chemiluminescent Substrate (Pierce, ThermoFisher Scientific, Waltham, MA, USA) following the manufacturer’s instructions, and chemiluminescence was detected using a CCD camera (Fusion FX, Vilber, Marne-la-Vallée, France). Band intensities were quantified by measuring their pixel density using ImageJ software to calculate a P-β-catenin/β-catenin relative ratio.

### 4.7. Statistical Analysis

SPSS^®^ Statistics version 23 software (IBM^®^, Armonk, NY, USA) was used for the statistical analysis. Normality was checked using the Shapiro-Wilk test, confirming the applicability of parametric analyses using the Student’s *t*-test. Data were expressed as means ± SD with a *p* ≤ 0.05 value to indicate a statistically significant difference.

## Data Availability

Data are contained within the article.

## References

[1] Cardiovascular Diseases. https://www.who.int/health-topics/cardiovascular-diseases.

[2] Cancer. https://www.who.int/news-room/fact-sheets/detail/cancer.

[3] Cancer (IARC) T.I.A. for R. on Global Cancer Observatory. https://gco.iarc.fr/.

[4] Faxon D.P., Fuster V., Libby P., Beckman J.A., Hiatt W.R., Thompson R.W., Topper J.N., Annex B.H., Rundback J.H., Fabunmi R.P. (2004). Atherosclerotic Vascular Disease Conference. Circulation.

[5] Gimbrone M.A., García-Cardeña G. (2016). Endothelial Cell Dysfunction and the Pathobiology of Atherosclerosis. Circ. Res..

[6] Rafieian-Kopaei M., Setorki M., Doudi M., Baradaran A., Nasri H. (2014). Atherosclerosis: Process, Indicators, Risk Factors and New Hopes. Int. J. Prev. Med..

[7] Kzhyshkowska J., Neyen C., Gordon S. (2012). Role of macrophage scavenger receptors in atherosclerosis. Immunobiology.

[8] Souilhol C., Harmsen M.C., Evans P.C., Krenning G. (2018). Endothelial–mesenchymal transition in atherosclerosis. Cardiovasc. Res..

[9] Tapia-Vieyra J.V. (2017). Atherosclerosis and Cancer; A Resemblance with Far-reaching Implications. Arch. Med. Res..

[10] Diakowska D., Grabowski K., Nienartowicz M., Zarębski P., Fudalej K., Markocka-Mączka K. (2015). Circulating oxidized low-density lipoproteins and antibodies against oxidized low-density lipoproteins as potential biomarkers of colorectal cancer. Gastroenterol. Res. Pract..

[11] Delimaris I., Faviou E., Antonakos G., Stathopoulou E., Zachari A., Dionyssiou-Asteriou A. (2007). Oxidized LDL, serum oxidizability and serum lipid levels in patients with breast or ovarian cancer. Clin. Biochem..

[12] Bitorina A.V., Oligschlaeger Y., Shiri-Sverdlov R., Theys J. (2019). Low profile high value target: The role of OxLDL in cancer. Biochim. Biophys. Acta BBA—Mol. Cell Biol. Lipids.

[13] Balzan S., Lubrano V. (2018). LOX-1 receptor: A potential link in atherosclerosis and cancer. Life Sci..

[14] Li C., Zhang J., Wu H., Li L., Yang C., Song S., Peng P., Shao M., Zhang M., Zhao J. (2017). Lectin-like oxidized low-density lipoprotein receptor-1 facilitates metastasis of gastric cancer through driving epithelial-mesenchymal transition and PI3K/Akt/GSK3β activation. Sci. Rep..

[15] Kindt N., Journé F., Carlier S., Trelcat A., Scalia A., Saussez S. (2021). Effect of Oxidized Low-Density Lipoprotein on Head and Neck Squamous Cell Carcinomas. Biomedicines.

[16] Shintani H. (2013). LDL Isolation and Copper-Catalysed Oxidation. Pharm. Anal. Acta.

[17] Li K., Wong D.K., Luk F.S., Kim R.Y., Raffai R.L. (2018). Isolation of Plasma Lipoproteins as a Source of Extracellular RNA. Extracellular RNA.

[18] Redgrave T.G., Roberts D.C.K., West C.E. (1975). Separation of plasma lipoproteins by density-gradient ultracentrifugation. Anal. Biochem..

[19] Aviram M. (1983). Plasma lipoprotein separation by discontinuous density gradient ultracentrifugation in hyperlipoproteinemic patients. Biochem. Med..

[20] Hirano T., Ito Y., Saegusa H., Yoshino G. (2003). A novel and simple method for quantification of small, dense LDL. J. Lipid Res..

[21] Galle J., Wanner C., Armstrong D. (1998). Oxidized LDL and Lp(a). Free Radical and Antioxidant Protocols.

[22] Seo J.-W., Yang E.-J., Yoo K.-H., Choi I.-H. (2015). Macrophage Differentiation from Monocytes Is Influenced by the Lipid Oxidation Degree of Low Density Lipoprotein. Mediat. Inflamm..

[23] Vlaminck B., Calay D., Genin M., Sauvage A., Ninane N., Zouaoui Boudjeltia K., Raes M., Michiels C. (2014). Effects of copper sulfate-oxidized or myeloperoxidase-modified LDL on lipid loading and programmed cell death in macrophages under hypoxia. Hypoxia.

[24] Reis D.R.A., Medeiros-Fonseca B., Costa J.M., de Oliveira Neto C.P., Gil da Costa R.M., Oliveira P.A., Medeiros R., Bastos M.M.S.M., Brito H.O., Brito L.M.O. (2020). HPV infection as a risk factor for atherosclerosis: A connecting hypothesis. Med. Hypotheses.

[25] Kuo H.-K., Fujise K. (2011). Human Papillomavirus and Cardiovascular Disease Among U.S. Women in the National Health and Nutrition Examination Survey, 2003 to 2006. J. Am. Coll. Cardiol..

[26] Lawson J.S., Glenn W.K., Tran D.D., Ngan C.C., Duflou J.A., Whitaker N.J. (2015). Identification of Human Papilloma Viruses in Atheromatous Coronary Artery Disease. Front. Cardiovasc. Med..

[27] Joo E.-J., Chang Y., Kwon M.-J., Cho A., Cheong H.S., Ryu S. (2019). High-Risk Human Papillomavirus Infection and the Risk of Cardiovascular Disease in Korean Women. Circ. Res..

[28] Miserocchi G., Cocchi C., De Vita A., Liverani C., Spadazzi C., Calpona S., Di Menna G., Bassi M., Meccariello G., De Luca G. (2021). Three-dimensional collagen-based scaffold model to study the microenvironment and drug-resistance mechanisms of oropharyngeal squamous cell carcinomas. Cancer Biol. Med..

[29] Engelmann L., Thierauf J., Koerich Laureano N., Stark H.-J., Prigge E.-S., Horn D., Freier K., Grabe N., Rong C., Federspil P. (2020). Organotypic Co-Cultures as a Novel 3D Model for Head and Neck Squamous Cell Carcinoma. Cancers.

[30] Vrieling F., Wilson L., Rensen P.C.N., Walzl G., Ottenhoff T.H.M., Joosten S.A. (2019). Oxidized low-density lipoprotein (oxLDL) supports Mycobacterium tuberculosis survival in macrophages by inducing lysosomal dysfunction. PLoS Pathog..

[31] Sparks D., Phillips M. (1992). Quantitative measurement of lipoprotein surface charge by agarose gel electrophoresis. J. Lipid Res..

[32] Schneider C.A., Rasband W.S., Eliceiri K.W. (2012). NIH Image to ImageJ: 25 years of image analysis. Nat. Methods.

[33] Kuda O., Pietka T.A., Demianova Z., Kudova E., Cvacka J., Kopecky J., Abumrad N.A. (2013). Sulfo-N-succinimidyl Oleate (SSO) Inhibits Fatty Acid Uptake and Signaling for Intracellular Calcium via Binding CD36 Lysine 164: SSO also inhibits oxidized low density lipoprotein uptake by macrophages. J. Biol. Chem..

[34] Sedgwick A.E., D’Souza-Schorey C. (2016). Wnt Signaling in Cell Motility and Invasion: Drawing Parallels between Development and Cancer. Cancers.

